# The association between sleep duration, bedtimes, and early pubertal timing among Chinese adolescents: a cross-sectional study

**DOI:** 10.1186/s12199-020-00861-w

**Published:** 2020-06-19

**Authors:** Hua Diao, Hong Wang, Lianjian Yang, Ting Li

**Affiliations:** School of Public Health and Management, Research Center for Medicine and Social Development, Collaborative Innovation Center of Social Risks Governance in Health, Chongqing Medical University, Chongqing, 400016 China

**Keywords:** Early pubertal timing, Sleep duration, Bedtimes, Adolescents

## Abstract

**Background and objective:**

Early pubertal timing is associated with sleep among Western adolescents, but little is known about this association in Chinese adolescents, especially with regard to the association between bedtimes and early pubertal timing. This paper aimed to identify the association between sleep duration, bedtimes, and early pubertal timing in Chinese adolescents.

**Methods:**

An anonymous cross-sectional survey was conducted among primary and junior middle students (grades 3 to 9) from QiJiang District, ChongQing, China. Participants were recruited by applying stratified cluster sampling. Pubertal timing, sleep duration, and bedtimes were assessed using the Pubertal Development Scale and a self-designed sleep questionnaire. We utilized multivariable logistic linear regression (MLLR) to test the association between sleep duration, bedtimes, and pubertal timing.

**Results:**

A total of 5461 adolescents were evaluated, with mean age and BMI values of 11.41 ± 2.05 and 18.03 ± 3.03, respectively, of whom 1257 (23.02%) were in early pubertal timing. In MLLR controlling for age, BMI, family economic status, and other covariates, sufficient sleep (*b* = − 0.214, *P* = 0.032, OR = 0.808, 95% CI 0.664–0.982) was negatively related to early pubertal timing, and later bedtime (*b* = 0.195, *P* < 0.001, OR = 1.215, 95% CI 1.104–1.338) was positively associated with early pubertal timing.

**Conclusion:**

Students with early pubertal timing had less sleep duration and later bedtimes, which may be the result of increased stress caused by physical and psychological changes. Therefore, more attention should be paid to pubertal health education for adolescents during puberty. Further longitudinal studies are needed to confirm the causality between sleep and early pubertal timing in Chinese adolescents.

## Background

Adolescents are in a critical transition from childhood to adulthood that involves a sequence of physiological changes, such as growth spurts, the emergence of secondary sexual characteristics, and the development of gonads and genitals. The initial time of physiological changes is called pubertal timing [1, 2]. Because of individual differences, pubertal timing can be divided into early, on time, and late compared with same-age and same-gender peers. Multiple studies have shown that the age at menarche and Tanner II breast development (B2) in developed countries is younger than that in the past [3–5]. Chinese children are also experiencing accelerated growth and advanced pubertal development, which has attracted the attention of Chinese experts [6–9]. In our previous studies, the average age at menarche in 2010 in urban areas of ChongQing was 0.26 years earlier than in 2005 [10]. The predominant determinant of early pubertal timing is genetic factors [11, 12], although the influence of environmental, social, and physical factors, such as gender, parental educational level, family structure, and body fat, are also indispensable [11, 13–17]. Early pubertal timing is not only associated with externalized behaviors such as aggression, smoking, drinking, and substance abuse but also closely related to internalized psychopathological symptoms such as anxiety, depression, and eating disorders [18, 19]. During adolescence, physiological and psychological upheavals are stressful events for children and adolescents [20], and early pubertal timing aggravates negative experiences such as conflicts between parents and children and conflicts among peers, resulting in difficulties in social adaptation and lower quality of life [21]. Therefore, early pubertal timing has attracted the attention of many researchers.

Sleep is crucial for adolescents’ pubertal development [22–24], and there is a significant correlation between circadian sleep and early pubertal timing [25, 26]. Hoyt et al. showed that the early development of breasts and pubic hair was related to sleep duration and bedtimes in black girls but not in Hispanic, white, and Asian girls [26]. Foley et al. indicated that the development of discrete aspects of secondary sex characteristics was uniquely associated with rhythm changes in sleep duration, eveningness preference, bedtimes, and wake times for girls but not for boys [25]. In China, some studies have found that sleep duration is relevant to the age and incidence of first spermatorrhea for boys and the incidence of menarche for girls [27–30], but few studies have explored the associations between bedtimes and early pubertal timing. Currently, there are few domestic or international studies on the relationship between sleep and youthfulness, and there are obvious individual and racial differences in both circadian sleep and pubertal timing. Therefore, the aims of this study are to identify the association between circadian sleep and early pubertal timing in Chinese adolescents. Our research presumes that insufficient sleep duration and later bedtimes increase the possibility of early pubertal timing in Chinese adolescents.

## Methods

### Design and setting

This research was a cross-sectional study. A multistage cluster sampling design was conducted to select 11 schools in QiJiang District, ChongQing. In stage 1, a better-cooperating district was selected in ChongQing. In stage 2, 6 primary schools and 5 junior middle schools were randomly chosen from this district. In stage 3, 6–8 classes were randomly selected from grades 3 to 6 in primary school and grades 7 to 9 in junior middle school. The selected 7446 students participated in our investigation, of whom 6991 (response rate 6991/7446 = 93.89%) completed a 40-min questionnaire without obvious logical mistakes or missing items. The study was approved by the ethical committee of ChongQing Medical University, and written informed consent was obtained from students and their parents before the research was conducted. Data collection occurred during 1 week in December 2017.

### Measures

1. Sociodemographic information
  Based on the influencing factors of pubertal timing in previous studies [11, 13–17], a self-designed questionnaire was applied to collect the following information: age, sex (male/female), whether there was only one child in the family (yes/no), parents’ education level (low: junior middle school or lower; medium: senior middle school and technical secondary school; high: college or higher), family economic status (poor, medium, good), parents’ relationship (poor, neutral, harmonious), and family parenting style (democratic: respectful, understanding, and supportive; autocratic: strict, with excessive behavioral regulation; doting; disregardful).
  BMI was calculated based on the formula weight in kilograms divided by height in meters squared. Height was accurate to within 0.1 cm, and weight was accurate to within 0.1 kg. Height and weight data were obtained from QiJiang Primary and Secondary School Students Health Care Center in ChongQing.
2. Pubertal timing
  Pubertal timing was assessed by the Pubertal Development Scale (PDS) from Petersen and Crockett [31] involving 5 items (common items in boys and girls: growth spurt, pubic hair growth, skin change—pimples; items in boys only: deepening voice change, facial hair; items in girls only: breast development, menarche). Every entry except the item menarche has four options: “not yet started (1 point),” “barely started (2 points),” “definitely started (3 points),” or “seems completed (4 points).” The remaining item, whether menarche occurred, involves two options: yes (4 points) or no (1 point). The average score of all items was used to assess pubertal timing (early, on time, late). In this study, children with a score of P75 or higher were defined as early pubertal timing, those with scores between P25 and P75 were defined as the control group (on time), and those with P25 or lower were defined as late pubertal timing [10, 32].
3. Sleep timing
  Sleep duration (SD) and bedtimes (BT) were obtained through the following questions: (Q1), what time do you go to sleep on weekdays and weekends (hour, minute, am/pm); (Q2), what time do you get up on weekdays and weekends (hour, minute, am/pm)? The intervals between Q1 and Q2 were defined as the sleep duration on weekdays (SD1) and weekends (SD2), respectively. Sleeping times were defined as bedtimes on weekdays (BT1) and weekends (BT2). Average sleep duration (ASD) and average bedtime (ABT) were calculated based on the following formulas: ASD = (SD1 × 5 + SD2 × 2)/7; ABT = (BT1 × 5 + BT2 × 2)/7. According to the standards of sleep duration recommended by the American Academy of Sleep Medicine in 2016 [33], students aged 6 to 12 years who slept less than 9 h and students aged 13 to 18 years who slept less than 8 h were classified into the “insufficient sleep group,” and students with sleep times longer than the above criteria were classified into the “sufficient sleep group.”

### Statistical analyses

Data collation and analysis were conducted using Epidata 3.0 and SPSS 21.0. First, according to the PDS scores, early (≥ P75) and on-time (P25–P75) pubertal timing were determined. Then, average sleep duration was categorized into a ternary variable with two categories, insufficient and sufficient, and bedtime was divided into two groups, ≤ 10 pm and > 10 pm. Chi-squared and *t* tests were employed to examine the unadjusted associations among average sleep duration, bedtime, and pubertal timing. Further multivariate analyses were conducted to examine whether average sleep duration and bedtime were associated with pubertal timing through multivariable logistic linear regression (MLLR) while controlling for covariates involving sex, only child, parents’ education level, economic status, parents’ relationship, parenting style, age, and BMI. In the multivariate analyses, all variables affecting pubertal timing were included, and all missing observations on any variables were excluded from the analysis. Any result with a *P* value less than 0.05 was considered statistically significant.

### Quality control

We tried investigation tools to identify any problems that might occur during the test. With the cooperation of local primary and secondary school health centers and permission from the headmaster, the investigation was implemented, trained investigators administered the survey in each class, and the investigation questionnaire was completed by every subject independently. All questionnaires were given to the students for completion and were then reviewed by the investigators immediately after completion to ensure that the students had completed the forms correctly.

## Results

### Subject characteristics

Among the 6991 subjects, the mean age and BMI were 11.41 ± 2.05 and 18.08 ± 3.30, respectively, the number of students with normal pubertal timing was 4204 (male/female 2223/1981), and the number of students with early pubertal timing was 1257 (boy/girl 666/591) (Table 1). Because of the analysis of the relationship between early pubertal timing and sleep quality, we removed the sample with late pubertal timing (1530). Among the students with early and normal pubertal timing, 2889 (52.9%) were male, 2572 (47.1%) were female, 1019 (18.7%) were the only child, 4442 (81.3%) were not the only child, 2760 (50.5%) lived in a family with medium economic status, 4575 (83.8%) came from families with harmonious parent relationships, and 3609 (66.1%) were raised in a democratic parenting style. With regard to the subjects’ mothers and fathers, 3209 (58.8%) and 3161 (57.9%) had a junior middle school or lower education, respectively.

**Table 1:** The scores of important puberty timing events in adolescents aged 8–15 in QiJiang District, Chongqing

Age	Male		Pubertal timing, *N*			Female		Pubertal timing, *N*		
	P25	P75	On time	Early	Late	P25	P75	On time	Early	Late
8	1.00	2.00	160	43	20	1.00	1.60	157	38	25
9	1.20	1.80	278	97	158	1.00	1.60	378	83	46
10	1.00	1.60	378	101	63	1.00	1.80	251	72	141
11	1.00	1.60	351	110	46	1.40	2.20	241	97	131
12	1.20	2.00	331	100	151	1.60	2.60	236	87	128
13	1.60	2.40	290	89	148	2.20	2.80	297	92	119
14	1.80	2.60	282	97	119	2.40	3.00	319	79	127
15	2.00	2.80	153	29	49	2.45	3.00	102	43	59
Total	--	--	2223	666	754	--	--	1981	591	776

Chi-squared and *t* tests revealed that insufficient sleep duration was more prevalent among students whose parents had less education (*P* < 0.001), students with poor economic status (*P* < 0.001) and parents’ relationship (*P* = 0.033), and students with older age (*P* < 0.001) or higher BMI (*P* < 0.001). The results of later bedtime were similar: the bedtimes of females (*P* < 0.001) and only children (*P* = 0.044) were later; students who were the only child (*P* = 0.004) and students with higher BMI (*P* < 0.001) were more likely to undergo early pubertal timing; and parenting style significantly affected sleep duration (*P* = 0.046), bedtime (*P* = 0.014), and pubertal timing (*P* = 0.031). All results are shown in Table 2.

**Table 2:** Characteristics of adolescents with regard to sleep duration, bedtime, and pubertal timing

Characteristics, *N* (%)	Sleep duration *X* (SD)/*N* (%)		P	Bedtime *X* (SD)/*N* (%)		*P*	Pubertal timing *X* (SD)/*N* (%)		*P*
	Insufficient	Sufficient		≤ 10 pm	> 10 pm		On time	Early	
Sex									
Male, 2889 (52.9)	438 (15.2)	2451 (84.8)	0.840	2253 (78.0)	636 (22.0)	< 0.001	2223 (76.9)	666 (23.1)	0.948
Female, 2572 (47.1)	395 (15.4)	2177 (84.6)		1894 (73.6)	678 (26.4)		1981 (77.0)	591 (23.0)	
Only child									
Yes, 1019 (18.7)	163 (16.0)	856 (84.0)	0.465	749 (73.5)	270 (26.5)	0.044	750 (73.6)	269 (26.4)	0.004
No, 4442 (81.3)	670 (15.1)	3772 (84.9)		3398 (76.5)	1044 (23.5)		3454 (77.8)	988 (22.2)	
Father’s education level									
Low, 3161 (57.9)	545 (17.2)	2616 (82.8)	< 0.001	2238 (70.8)	923 (29.2)	< 0.001	2408 (76.2)	753 (23.8)	0.118
Medium, 2108 (28.6)	262 (12.4)	1846 (87.6)		1741 (82.6)	367 (17.4)		1653 (78.4)	455 (21.6)	
High, 192 (3.5)	26 (13.5)	166 (86.5)		168 (87.5)	24 (12.5)		143 (74.5)	49 (25.5)	
Mother’s education level									
Low, 3209 (58.8)	558 (17.4)	2651 (82.6)	< 0.001	2249 (70.1)	960 (29.9)	< 0.001	2451 (76.4)	758 (23.6)	0.297
Medium, 2084 (38.2)	244 (11.7)	1840 (88.3)		1748 (83.9)	336 (16.1)		1627 (78.1)	457 (21.9)	
High, 168 (3.0)	31 (18.5)	137 (81.5)		150 (89.3)	18 (10.7)		126 (75.0)	42 (25.0)	
Economic status									
Poor, 719 (13.2)	120 (16.7)	599 (83.3)	< 0.001	525 (73.0)	194 (27.0)	< 0.001	547 (76.1)	172 (23.9)	0.168
Medium, 2760 (50.5)	472 (17.1)	2288 (82.9)		1915 (69.4)	845 (30.6)		2103 (76.2)	657 (23.8)	
Good, 1982 (36.3)	241 (12.2)	1741 (87.8)		1707 (86.1)	275 (13.9)		1554 (78.4)	428 (21.6)	
Parents’ relationship									
Poor, 177 (3.2)	38 (21.5)	139 (78.5)	0.033	125 (70.6)	52 (29.4)	0.004	138 (78.0)	39 (22.0)	0.195
Neutral, 709 (13.0)	117 (16.5)	592 (83.5)		509 (71.8)	200 (28.2)		527 (74.3)	182 (25.7)	
Harmonious, 4575 (83.8)	678 (14.8)	3897 (85.2)		3513 (76.8)	1066 (23.2)		1539 (77.4)	1036 (22.6)	
Parenting style									
Democratic, 3609 (66.1)	567 (15.7)	3042 (84.3)	0.046	2717 (75.3)	892 (24.7)	0.014	2794 (77.4)	815 (22.6)	0.031
Autocratic, 1012 (18.5)	164 (16.2)	848 (83.8)		756 (74.7)	256 (25.3)		773 (76.4)	239 (23.6)	
Doting, 601 (11.0)	70 (11.6)	531 (88.4)		486 (80.9)	115 (19.1)		471 (78.4)	130 (21.6)	
Disregardful, 239 (4.4)	32 (13.4)	207 (86.6)		188 (78.7)	51 (21.3)		166 (69.5)	73 (30.5)	
Age	12.07 (1.74)	11.30 (2.07)	< 0.001	10.83 (1.88)	13.26 (1.33)	< 0.001	11.40 (2.06)	11.47 (1.99)	0.281
BMI	18.90 (3.36)	17.94 (3.27)	< 0.001	17.66 (3.30)	19.43 (2.92)	< 0.001	17.94 (3.26)	18.57 (3.39)	< 0.001

### Association between sleep and pubertal timing

Chi-squared tests showed that students with early pubertal timing were more susceptible to insufficient sleep duration (*P* < 0.001) and later bedtimes (*P* < 0.001) (Table 3).

**Table 3:** Unadjusted analyses between sleep duration, bedtime, and pubertal timing

Sleep, *N* (%)	Pubertal timing N (%)		*χ*^2^	*P*
	On time	Early		
Sleep duration				
Insufficient, 833 (15.3)	580 (69.6)	253 (30.4)	30.003	< 0.001
Sufficient, 4628 (84.7)	3624 (78.3)	1004 (21.7)		
Bedtime groups				
≤ 10 pm, 4147 (75.9)	3246 (78.3)	901 (21.7)	16.216	< 0.001
> 10 pm, 1314 (24.1)	958 (72.9)	356 (27.1)		

MLLR was conducted to further explore the association controlling for covariates involving sex, only child, parents’ education level, economic status, parents’ relationship, parenting style, age, and BMI. MLLR (Table 4) revealed that sufficient sleep duration (*b* = − 0.214, *P* = 0.032, OR = 0.808, 95% CI 0.664–0.982) was negatively related to early pubertal timing, and later bedtime (*b* = 0.195, *P* < 0.001, OR = 1.215, 95% CI 1.104–1.338) was positively associated with early pubertal timing.

**Table 4:** Standardized coefficients obtained from multiple logistic linear regression (MLLR)

Independent variable(s)	MLLR (*N* = 5461)			
	β	SE	P	OR (95% CI)
Sleep duration				
Insufficient	Ref			
Sufficient	− 0.214	0.100	0.032	0.808 (0.664–0.982)
Bedtimes (pm)^a^	0.195	0.049	< 0.001	1.215 (1.104–1.338)
Sex				
Male	Ref			
Female	− 0.014	0.066	0.828	0.986 (0.867–1.121)
Only child				
Yes	Ref			
No	− 0.224	0.081	< 0.001	0.800 (0.682–0.937)
Father’s education level				
Low	Ref			
Medium	− 0.092	0.083	0.267	0.912 (0.775–1.073)
High	0.145	0.194	0.452	1.157 (0.791–1.690)
Mother’s education level				
Low	Ref			
Medium	− 0.028	0.083	0.735	0.972 (0.826–1.144)
High	− 0.009	0.209	0.996	0.991 (0.658–1.492)
Economic status				
Poor	Ref			
Medium	0.005	0.100	0.958	1.005 (0.826–1.224)
Good	− 0.080	0.109	0.462	0.923 (0.745–1.143)
Parents’ relationship				
Poor	Ref			
Neutral	0.273	0.204	0.180	1.314 (0.881–1.959)
Harmonious	0.185	0.190	0.332	1.203 (0.828–1.747)
Parenting style				
Democratic	Ref			
Autocratic	0.035	0.087	0.689	1.035 (0.873–1.228)
Doting	− 0.052	0.110	0.636	0.949 (0.765–1.178)
Disregardful	0.143	0.152	0.007	1.512 (1.122–2.036)
Age	− 0.096	0.023	< 0.001	0.909 (0.869–0.951)
BMI	0.056	0.010	< 0.001	1.057 (1.037–1.078)

The dependent variable was pubertal timing: on-time = 0; early pubertal timing = 1

^a^Bedtimes as a continuous variable

Regarding the students’ characteristics, early pubertal timing was more prevalent in students with higher BMI (*b* = 0.056, *P* < 0.001, OR = 1.057, 95% CI 1.037–1.078) and those with a disregardful parenting style (*b* = 0.143, *P* = 0.007, OR = 1.512, 95% CI 1.122–2.036). However, children with biological brothers or sisters (*b* = − 0.224, *P* < 0.001, OR = 0.800, 95% CI 0.682–0.937) and older students (b = − 0.096, *P* < 0.001, OR = 0.909 95% CI 0.869–0.951) had less risk of early pubertal timing according to the MLLR (Table 4).

## Discussion

Sufficient sleep is essential for adolescents’ physical and mental well-being during adolescence. However, 66–92% of adolescents do not get the recommended 8–10 h of sleep in the evening [34, 35]. Insufficient sleep can have a huge negative impact on numerous adverse academic, safety, mental health, and physical health outcomes [36–38]. Our results indicated that insufficient sleep and later bedtime were more prevalent among students whose parents had less education. Parents with a high degree of education are more likely to obtain sleep-related health knowledge through books, newspapers, and the Internet to help adolescents have healthy sleep [39]. The findings also showed that students with poor economic status and poor parent relationships were more susceptible to insufficient sleep duration and later bedtimes. The reason may be that a good family economy and harmonious parental relationship may create a good living environment for adolescents, thus reducing the incidence of negative life events and improving sleep quality. Additionally, the study found that individuals with older age or higher BMI had more insufficient sleep duration and later bedtimes. As adolescents become older, academic pressures increase, leading to sleep loss and later bedtime. Furthermore, overweight or obesity is a risk factor for many sleep breathing diseases [40, 41], which is consistent with our findings. The research also suggested that later bedtimes were predominant in only children. A previous study found that because the family is the initial living environment, parents have a profound role in children’s sleep habits. Parents of only children, compared with those with multiple children, pay excessive attention to their child’s sleep habits, leading to a greater susceptibility to the personality traits of cowardice and diffidence; these negative moods may result in poor sleep habits and sleep disorders [42].

Moreover, we found that students with early pubertal timing had less sleep duration and later bedtimes compared to their counterparts with on time puberty in the MLLR analysis, which is in accordance with reported findings in previous studies [25–28]. For instance, a study conducted by Foley et al. showed that girls with late bedtimes on weekdays had earlier pubic hair development and faster breast development during puberty [25]. In another study, Hoyt et al. found that early pubertal timing in girls was related to shorter sleep duration, but there was no association with pubertal rate [26]. In China, Wen et al. found that insufficient sleep for boys increased the probability of first spermatorrhea [27]. The reasons for shorter sleep duration and later bedtimes among students with early pubertal timing involve two aspects: sociopsychological factors and hormone secretion. First, the onset of puberty, which involves a sequence of physical and psychological upheavals, is a stressful event [20] that may lead to parental conflicts and peer conflicts. Earlier pubertal timing exacerbates these stresses [43, 44], giving rise to internalizing problems such as depression and anxiety [18, 19]. Furthermore, sleep duration and betimes may be influenced negatively. Second, increases in estrogen and insulin-growth factor 1 (IGF1) can promote the development of the breast and pubic hair, and adrenal androgens can stimulate the growth of pubic hair. This information indicates that maturation of the adrenal glands may negatively impact sleep duration and bedtime [25, 45]. Similarly, after adolescents enter puberty, their melatonin secretion time changes, leading to delays in sleep time and wakefulness [27]. A study conducted by Zwart et al. [46] showed that long-term melatonin treatment in adolescents with chronic sleep disorders can improve sleep quality and delay puberty initiation.

The association between less sleep duration, later bedtimes, and early pubertal timing can be recognized in the wider context of the influencing factors of pubertal timing. The data analysis indicated that the students with higher BMI have a higher risk of undergoing early pubertal timing, which is similar to previous studies [14, 15]. Insulin resistance in obese students decreases the level of sex hormone-binding protein in the liver, which increases estrogen levels and promotes pubertal development [15]. Earlier pubertal timing was prevalent among subjects with a disregardful parenting style, which is supported by Wang et al. [47]. This may be due to the lack of parental care, resulting in increased family pressure. Subjects with biological brothers or sisters were less sensitive to earlier pubertal timing. When these individuals face pubertal changes and stress during adolescence, they can communicate with their brothers or sisters to relieve stress. Younger students experienced earlier pubertal timing, which is inconsistent with the findings of Wen et al. [27]. The reasons for this divergence may be that they explored the association between the incidence of first spermatorrhea and age, and there was no clear definition of earlier first spermatorrhea.

## Limitation

This research is a preliminary exploration of the association between sleep and early pubertal timing in Chinese adolescents, and as such, there are several limitations that should be considered in the interpretation of the results. First, the subjects in our study were from 11 selected schools in one district in Chongqing, which may not represent the whole population of adolescents. Furthermore, the study was a cross-sectional study, and we could not establish causality. Finally, sleep duration only involved sleep duration at night, which may have had an impact on the final results. Therefore, further studies are necessary to better detect the association between sleep and early pubertal timing.

## Conclusions and implications

This study suggests that students with early pubertal timing have less sleep duration and later bedtimes. This may be the result of increased stress caused by physical and psychological changes. Therefore, more attention should be paid to pubertal health education for adolescents during puberty. Additionally, further longitudinal studies are needed to confirm the causality between sleep and early pubertal timing in Chinese adolescents.

## Supplementary information

**Additional file 1.** Approval notice of research paper.

**Additional file 2.** Analysis in both sexes (boys and girls).

**Additional file 3.** Relationship between parenting style and only child, sleep duration.

## Data Availability

All data generated or analyzed during this study are included in this published article and its supplementary documents.

## Abbreviations

SD: Sleep duration
BT: Bedtime
ASD: Average sleep duration
ABT: Average bedtime
MLLR: Multivariable logistic linear regression
GLLM: Generalized linear mixed model
