# Supplementary figures and images for: The association between sleep duration, bedtimes, and early pubertal timing among Chinese adolescents: a cross-sectional study
Source: Environ Health Prev Med. 2020 Jun 19;25:21. doi: 10.1186/s12199-020-00861-w (PMC7305621; doi:10.1186/s12199-020-00861-w)

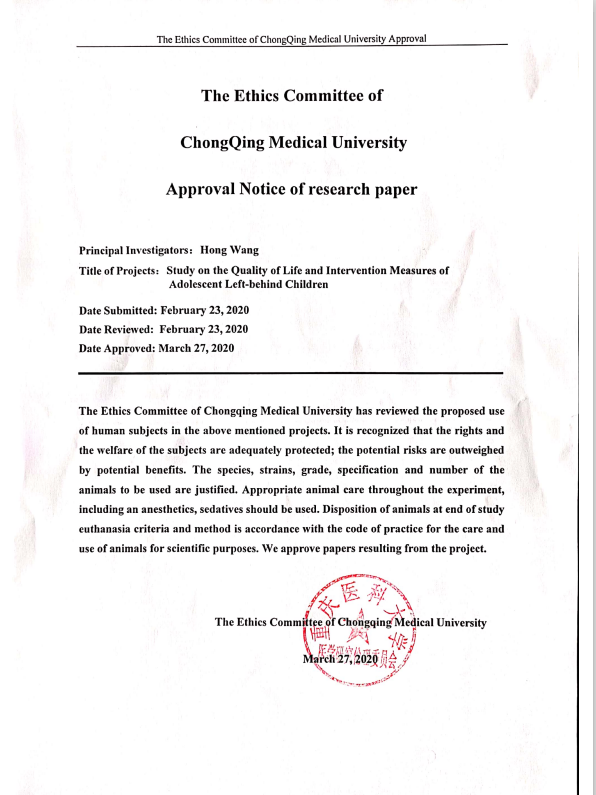

Supplement: Supplementary file 1 — Additional file 1. Approval notice of research paper. [file 12199_2020_861_MOESM1_ESM.doc]
