# Supplementary material for: The association between sleep duration, bedtimes, and early pubertal timing among Chinese adolescents: a cross-sectional study
Source: Environ Health Prev Med. 2020 Jun 19;25:21. doi: 10.1186/s12199-020-00861-w (PMC7305621; doi:10.1186/s12199-020-00861-w)
Supplement: Supplementary file 2 — Additional file 2. Analysis in both sexes (boys and girls). [file 12199_2020_861_MOESM2_ESM.doc]

| Table 1 The relation between sleep duration, bedtime and pubertal timing in boys and girls | | | | | |
| --- | --- | --- | --- | --- | --- |
| **Sex, n(%)** | **Sleep, n(%)** | **Pubertal timing, n(%)** | | ***^χ2^*** | ***P*** |
|  |  | **On time** | **Early** |  |  |
| **Boys 2889(52.9)** | **Sleep duration** |  |  |  |  |
|  | Insufficient, 307(10.6) | 214(69.7) | 93(30.3) | 10.151 | 0.001 |
|  | Sufficient, 2582(89.4) | 2009(77.8) | 573(22.2) |  |  |
|  | **Bedtime** |  |  |  |  |
|  | ≤10pm, 2253(78.0) | 1758(78.0) | 495(22.0) | 6.758 | 0.010 |
|  | >10pm, 636(22.0) | 465(73.1) | 171(26.9) |  |  |
| **Girls 2572(47.1)** | **Sleep duration** |  |  |  |  |
|  | Insufficient, 248(9.6) | 179(72.2) | 69(27.8) | 3.639 | 0.056 |
|  | Sufficient, 2324(90.4) | 1802(77.5) | 522(22.5) |  |  |
|  | **Bedtime** |  |  |  |  |
|  | ≤10pm, 1894(73.6) | 1488(78.6) | 406(21.4) | 9.654 | 0.002 |
|  | >10pm, 678(26.4) | 493(72.7) | 185(27.3) |  |  |
