# Supplementary material for: The association between sleep duration, bedtimes, and early pubertal timing among Chinese adolescents: a cross-sectional study
Source: Environ Health Prev Med. 2020 Jun 19;25:21. doi: 10.1186/s12199-020-00861-w (PMC7305621; doi:10.1186/s12199-020-00861-w)
Supplement: Supplementary file 3 — Additional file 3. Relationship between parenting style and only child, sleep duration. [file 12199_2020_861_MOESM3_ESM.doc]

| table 2 The relation between only child and parenting styles | | | | |
| --- | --- | --- | --- | --- |
| **Only child** | **Autocratic and doting parenting styles, n(%)** | | ***χ2*** | ***P*** |
|  | **No** | **Yes** |  |  |
| **Yes** | 690(67.7) | 329(32.3) | 4.552 | 0.033 |
| **No** | 3158(71.1) | 1284(28.9) |  |  |

| Table 3 The logistic regression between sleep duration and parenting styles | | | | |
| --- | --- | --- | --- | --- |
| Independent variable(s) | MLLR(N=5461) | | | |
|  | b(SE) | SE | P | OR(95%CI) |
| Autocratic and doting parenting styles | | | | |
| No | Ref |  |  |  |
| Yes | －0.247 | 0.099 | 0.012 | 0.781(0.644,0.948) |
| Sex | | | | |
| Male | Ref |  |  |  |
| Female | 0.082 | 0.092 | 0.369 | 1.086(0.907,1.300) |
| Father's education level | | | | |
| Low | Ref |  |  |  |
| Medium | －0.051 | 0.116 | 0.659 | 0.950(0.756,1.193) |
| High | －0.257 | 0.265 | 0.331 | 0.773(0.460,1.299) |
| Mother's education level | | | | |
| Low | Ref |  |  |  |
| Medium | －0.005 | 0.117 | 0.963 | 0.995(0.790,1.252) |
| High | －0.616 | 0.246 | 0.02 | 0.540(0.322,0.907) |
| Economic status | | | | |
| Poor | Ref |  |  |  |
| Medium | －0.046 | 0.137 | 0.737 | 0.955(0.731,1.248) |
| Good | －0.141 | 0.151 | 0.348 | 0.868(0.646,1.166) |
| Age | －0.246 | 0.026 | <0.001 | 0.782(0.744,0.823) |
| note:dependent variable:sleep duration,insufficient=0, sufficient=1 | | | | |
